# CRISPR/Cas9-mediated mutagenesis of *CAROTENOID CLEAVAGE DIOXYGENASE8* in tomato provides resistance against the parasitic weed *Phelipanche aegyptiaca*

**DOI:** 10.1038/s41598-019-47893-z

**Published:** 2019-08-07

**Authors:** Vinay Kumar Bari, Jackline Abu Nassar, Sally Marzouk Kheredin, Amit Gal-On, Mily Ron, Anne Britt, Daniel Steele, John Yoder, Radi Aly

**Affiliations:** 1 Department of Plant Pathology and Weed Research, Newe Ya’ar Research Center, Agricultural Research Organization (ARO), Volcani Center, Ramat Yishay, Israel; 2 Department of Plant Pathology and Weed Research, Agricultural Research Organization (ARO), Volcani Center, Bet-Dagan, Israel; 3 Department of Plant Biology and Genome Center, University of California, Davis, USA; 4 Department of Plant Sciences, University of California, Davis, USA

**Keywords:** Plant molecular biology, Molecular engineering in plants

## Abstract

Broomrapes (*Phelipanche aegyptiaca* and *Orobanche* spp.) are obligate plant parasites that cause extreme damage to crop plants. The parasite seeds have strict requirements for germination, involving preconditioning and exposure to specific chemicals strigolactones [SLs] exuded by the host roots. SLs are plant hormones derived from plant carotenoids via a pathway involving the Carotenoid Cleavage Dioxygenase 8 (*CCD8)*. Having no effective means to control parasitic weeds in most crops, and with CRISPR/Cas9 being an effective gene-editing tool, here we demonstrate that CRISPR/Cas9-mediated mutagenesis of the *CCD8* gene can be used to develop host resistance to the parasitic weed *P*. *aegyptiaca*. Cas9/single guide (sg) RNA constructs were targeted to the second exon of *CCD8* in tomato (*Solanum lycopersicum L*.) plants. Several ^*CCD8*^Cas9 mutated tomato lines with variable insertions or deletions in *CCD8* were obtained with no identified off-targets. Genotype analysis of T1 plants showed that the introduced *CCD8* mutations are inherited. Compared to control tomato plants, the ^*CCD8*^Cas9 mutant had morphological changes that included dwarfing, excessive shoot branching and adventitious root formation. In addition, SL-deficient ^*CCD8*^Cas9 mutants showed a significant reduction in parasite infestation compared to non-mutated tomato plants. In the ^*CCD8*^Cas9 mutated lines, orobanchol (SL) content was significantly reduced but total carotenoids level and expression of genes related to carotenoid biosynthesis were increased, as compared to control plants. Taking into account, the impact of plant parasitic weeds on agriculture and difficulty to constitute efficient control methods, the current study offers insights into the development of a new, efficient method that could be combined with various collections of resistant tomato rootstocks.

## Introduction

The clustered regularly interspaced short palindromic repeats/CRISPR-associated protein 9 (CRISPR/Cas9) system has emerged as a powerful genome-engineering technology with success in diverse organisms^1^. Cas9-mediated genome editing technology provides enormous advantages over other classical methods in crop improvement and plant research by generating desired modifications at a specific target sequence^2^. In some cases, CRISPR/Cas9 permits the direct introduction of mutations conferring resistance in crop plants, without traditional backcrosses or plant breeding^3^. Cas9-DNA scissors makes site-specific double-strand cut in the genome, inducing modifications at targeted locus through homologous recombination or non-homologous end-joining repair mechanisms while Cas9 base editor have ability to alter a specific nucleotide into another^4,5^. The most frequently used CRISPR/Cas9 system, type II, has three components: Cas9 endonuclease, CRISPR RNA (crRNA) and trans activating crRNA (tracrRNA). Cas9-mediated DNA cleavage is guided by a tracrRNA:crRNA duplex that is complementary to the target DNA^6^. Recently the tracrRNA:crRNA complex is fused into a single chimeric RNA known as single guide RNA (sgRNA) containing an 18 to 20-nucleotide (nt) sequence which determines the target DNA sequence^7^. The NGG protospacer adjacent motif (PAM) that is present at 3′-end of the target sequence was recognized by the CRISPR/Cas9 system^8^. Use of CRISPR/Cas9 has been reported as a most effective tool for nucleotide sequence modification or editing in numerous crop species, including Arabidopsis, wheat, rice, sorghum, cotton, maize, soybean and tobacco^9,10^.

The genera of parasitic weeds, *Orobanche* and *Phelipanche* (Orobanchaceae), the broomrapes, consist of over 100 species and represent one of the most destructive and great challenge in agricultural production. These are obligate plant parasites that attack through the host roots of almost all economically important crops in the Solanaceae, Fabaceae, Asteraceae, Brassicaceae and Apiaceae plant families^11,12^. The life cycle of *P*. *aegyptiaca* is divided into two stages, preparasitic and parasitic. The preparasitic stage consists of seed preconditioning followed by germination. The germination of parasite seeds is triggered by a highly specialized detection system for strigolactones (SLs) exuded by host roots^13,14^. The parasitic stage initiates with the parasite developing a special intrusive organ the haustorium- that connects directly to the vascular system of the host^15^. Following successful attachment and invasion of the host root, the broomrape seedling grows into a structure known as tubercle and after 4–5 weeks of tubercle growth, a floral meristem is produced, which emerges above the ground to produce flower and seeds.

SL is a plant hormone required for shoot branching and used as a signaling molecule for the rhizosphere microflora^16^. SLs occur in all green lineages of the plant kingdom and its synthesis start with the all trans β-carotene, a carotenoid molecule which produce 9-cis-β-carotene by the activity of Dwarf 27 (*D27*), after that Carotenoid Cleavage Dioxygenases 7 (*CCD7*) convert it into 9 cis β–apo 10′-carotenal and finally Carotenoid Cleavage Dioxygenases 8 (*CCD8*) leads to the production of carlactone, then cytochrome P450 enzymes, More Axillary Growth 1 (*MAX1*) convert it into various SLs^17^. Existence of homologs *CCD7* and *CCD8* have been reported in *P*. *ramosa* and *P*. *aegyptiaca*^18^. In the rhizosphere, SL acts as a host-detection cue for symbiotic arbuscular mycorrhizal fungi and stimulates seed germination of parasitic plants^19^. Different types of SLs, e.g., strigol, 5-deoxystrigol, sorgolactone, solanacol, didehydroorobanchol, orobanchol and others, are known as germination stimulants for root parasites^20^. The altered SL production conferred resistance in the host by reducing the germination of parasite seeds. Host resistance to the Orobanchaceae root parasite *Striga* has been observed in crops with altered SL production^21,22^. In addition, previous studies found that the tomato *SL-ORT1* mutagenized by fast-neutron display highly resistant to *Phelipanche* and *Orobanche spp*, the resistance results from its inability to produce and secrete SLs regarded as natural germination stimulants to the rhizosphere^23^. Different methods of parasitic weed-control (chemical, biological, cultural and resistant crops) have been applied in attempts to control broomrape^24^, but difficulties are encountered in targeting specific plant–plant systems. Moreover, most control strategies are less effective and have considerable limitations. Here we report the development of tomato (*Solanum lycopersicum L*.) plants that are resistant to the parasitic weed *P*. *aegyptiaca* upon mutation of the SL-biosynthesis gene *CCD8* using CRISPR/Cas9.

## Results

### CRISPR/Cas9-mediated editing of the tomato *CCD8* gene

**Figure 1:**
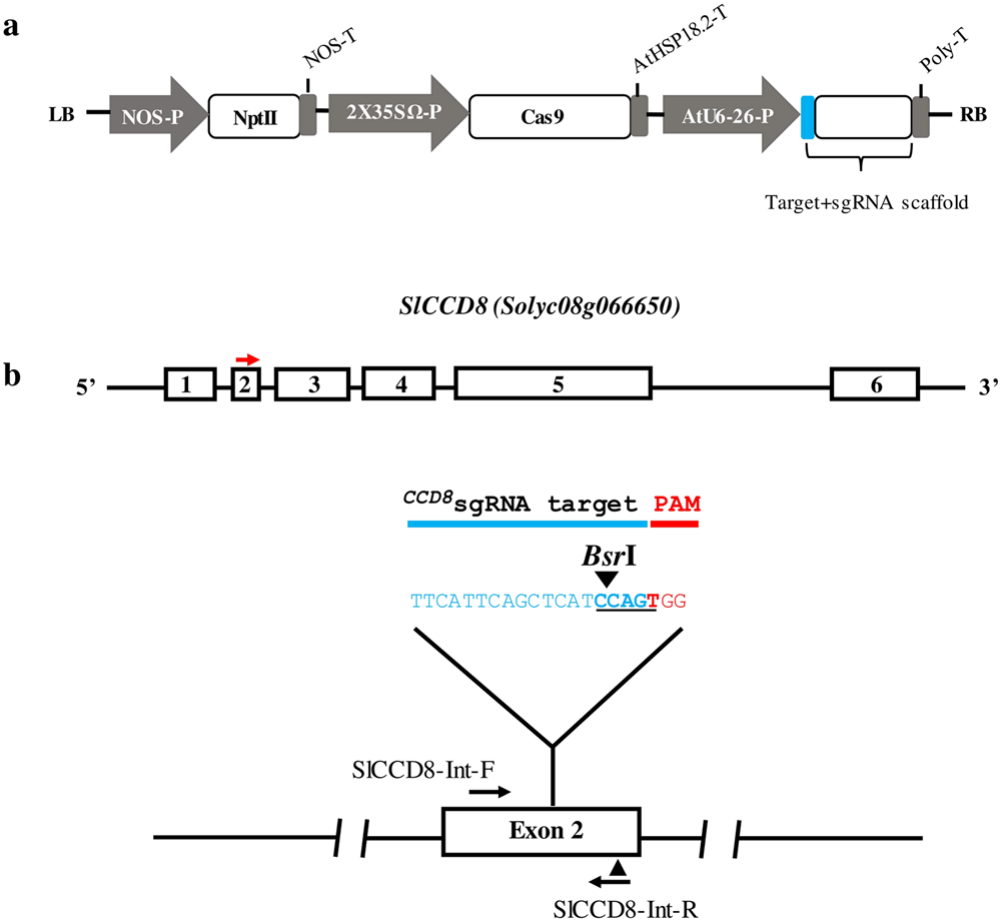
Schematic representation of the binary plant vector construct used for Cas9 and ^*CCD8*^sgRNA expression and *CCD8* locus. (**a)** The strong constitutive 2 × 35 SΩ promoter (CaMV-2 × 35S promoter with the omega enhancer sequence) was used to express *Arabidopsis* codon-optimized Cas9 and the *Arabidopsis* U6-26 promoter was used to express ^*CCD8*^sgRNA. **(b)** Schematic representation of the tomato *SlCCD8* genomic map and location of the ^*CCD8*^sgRNA target site. The target sequence of ^*CCD8*^sgRNA is shown in cyan color, the PAM is shown in red, the *Bsr*I restriction site with sequence is marked by a black triangle and underlined, black arrows indicate the primer positions (SlCCD8-Int-F and SlCCD8-Int-R) used for PCR amplification, and red arrows indicate location of ^*CCD8*^sgRNA target site.

**Figure 2:**
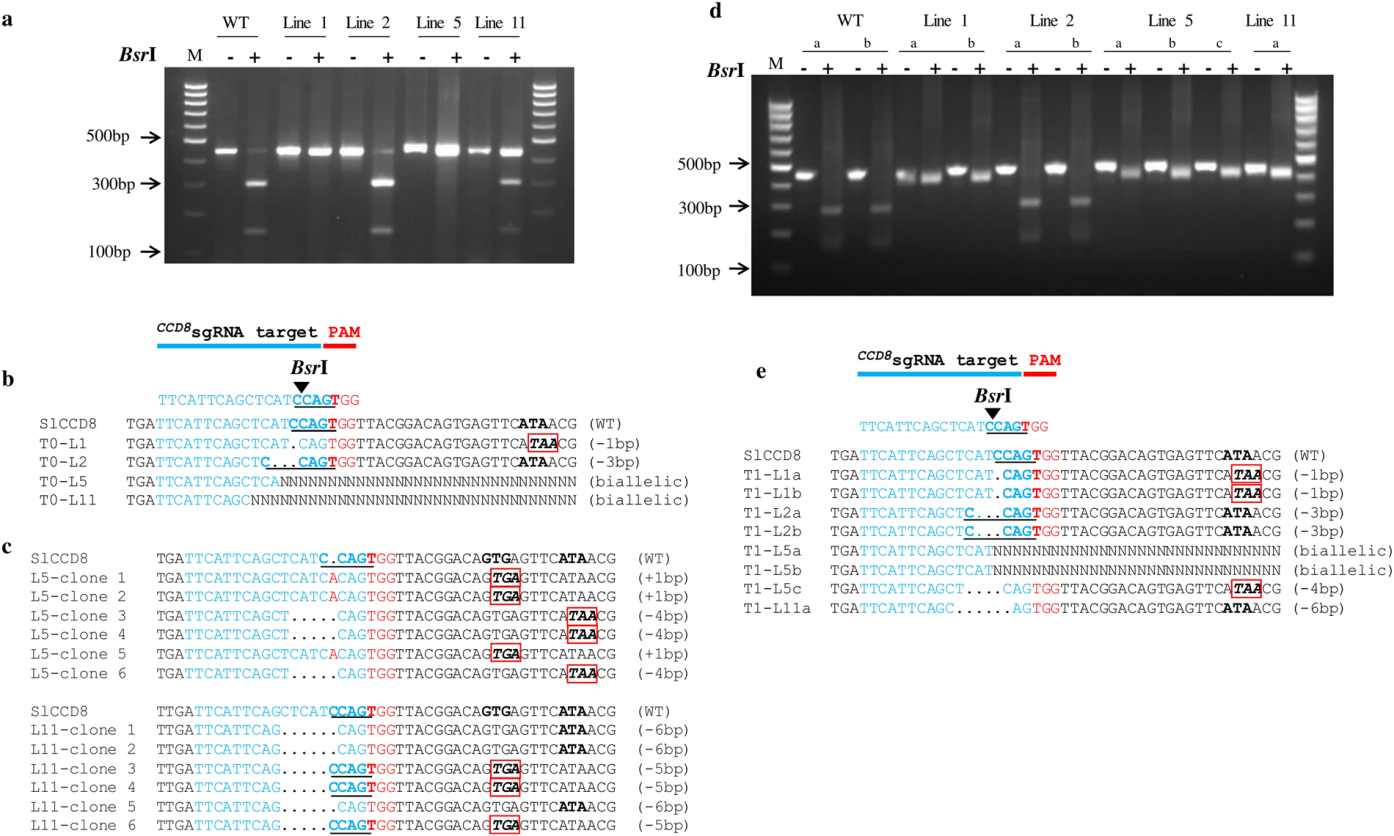
Restriction analysis and sequence alignment of ^*CCD8*^Cas9-edited lines. (**a**) PCR fragments of ^*CCD8*^Cas9 targeted region in T0 lines from genomic DNA of independent transgenic plants were subjected to *Bsr*I-restriction digestion. Lines 1, 2, 5 and 11 represent independent T0 transgenic plants;+, *Bsr*I added; −, without *Bsr*I. *Bsr*I digested or non-digested PCR product were resolved using a 2% agarose gel for 60 min. Results shown that ^*CCD8*^Cas9 mutant line 1 and 5 lost the *Bsr*I digestion site, while line 11 shown partial digestion, however line 2 yield bands similar to control wild-type (WT). The full length agarose gel blot represents the complete set of digested or non-digested PCR products run in independent wells of a single gel. Image was acquired using DNR MiniLumi with UV light and Microsoft office was used to crop the image in appropriate size. M: DNA size ladder (100 bp) **(b)** PCR product sequence alignment of the selected T0 lines with the wild-type genome sequences (WT). PAM is shown in red; *Bsr*I site is shown in bold underlined, black triangle indicates the digestion site for *Bsr*I; DNA deletions are shown in black dots and deletion sizes (nt) are marked on the right side of the sequences. **(c)** ^*CCD8*^Cas9 target regions from T0 lines 5 and 11 were amplified by PCR and cloned into the TA cloning vector. Sanger sequencing of positive clones aligned with wild-type sequence, and type of mutation (indels) detected presented on the right side of the sequences. Nucleotide sequence inside red box encode for stop codon. **(d)** PCR fragments of ^*CCD8*^Cas9 targeted region in T1 generation from genomic DNA of independent transgenic plants was subjected to *Bsr*I restriction digestion;+, *Bsr*I added; −, without *Bsr*I. *Bsr*I digested or non-digested PCR products were resolved using a 2.5% agarose gel for 60 min. Results shown that ^*CCD8*^Cas9 mutant line 1, 5 and 11 lost the *Bsr*I digestion site, however line 2 yield bands similar to control wild-type (WT). The full length agarose gel blot represents the complete set of digested or non-digested PCR products run in independent wells of a single gel. Image was acquired using DNR MiniLumi with UV light and Microsoft office was used to crop the image in appropriate size. M: DNA size ladder (100 bp) **(e)** PCR product sequence alignment of the selected T1 lines. PAM is shown in red; *Bsr*I site is shown in bold underlined, black triangle indicates digestion site for *Bsr*I; DNA deletions are shown in black dots and deletion sizes (nt) are marked on the right side of the sequences; Nucleotide sequence inside red box encode for stop codon.

To investigate the efficacy of using CRISPR/Cas9 to create host resistance in tomato plants against parasitic weeds, we chose to disrupt the SL-biosynthesis gene *CCD8* (*Solyc08g066650*) in tomato. SLs are synthesized from plant carotenoids via a pathway involving *CCD7* and *CCD8*. The ^*CCD8*^sgRNA construct was designed using the single sgRNA cassette in the pENTR vector, which was then recombined into the pDest vector to target the second exon of *CCD8* (position 716–733 bp in the coding region) with a *Bsr*I restriction site located upstream to the protospacer adjacent motif (PAM) (Fig. 1a,b). Fourteen independent T0 transgenic tomato lines were generated by *Agrobacterium*-mediated transformation at the Plant Transformation Facility of the University of California, Davis, USA. However, 10 out of 14 (71.4%) of the ^*CCD8*^Cas9 transgenic plants showed mutation at *SlCCD8* locus, as determined by PCR and restriction analysis (data not shown). Mutants showed similar kind of mutation at genome level considered as one single line in our study. We chose to continue our study with only those lines that showed different kind of genome editing in the T0 generation such as line 1, 2, 5 and 11 and the presence of transgene Npt-II was confirmed by PCR (Supplementary Fig. S1a).To detect mutations induced by the Cas9 nuclease in T0 plants, we assessed loss of the *Bsr*I restriction enzyme site that might arise due to imprecise non-homologous end-joining repair. For each of the T0 plants, we PCR-amplified the ^*CCD8*^Cas9 target region using genomic DNA from the T0 lines (individual transformants) and then digested it with *Bsr*I (a site that would disrupted if Cas9-mediated genome editing had occurred in this location) and examined products on a 2% agarose gel. Digestion of the wild-type PCR fragment (452 bp) with *Bsr*I results in three products of 147 bp, 298 bp and 7 bp, while Cas9 editing of the target site will generally eliminate the *Bsr*I site that is adjacent to the PAM, leading to a 445 bp fragment. Restriction digestion of the target region-specific PCR product from lines 1, 2, 5 and 11 revealed that lines 1 and 5 were fully resistant to *Bsr*I digestion, whereas line 11 showed partial digestion. However, line 2 showed complete digestion of the PCR product, similar to the wild type (Fig. 2a). Sequencing analysis of the target region PCR product from T0 lines 1, 2, 5 and 11 revealed the presence of different kinds of mutations in each line. Line 1 had a 1-nt deletion and line 2 had a 3-nt deletion; the 3-nt deletion in line 2 regenerated the *Bsr*I restriction site, resulting in *Bsr*I digestion of the PCR product in this line. In addition, these lines showed a single peak in the Sanger sequencing chromatogram, suggesting that they represent homozygous mutants for the ^*CCD8*^Cas9 locus (Fig. 2b). However, lines 5 and 11 showed multiple peaks in the sequencing chromatogram, suggesting that they are either biallelic or chimeric mutants (Supplementary Fig. S1b). Purified non-digested ^*CCD8*^Cas9 target region PCR fragments from lines 5 and 11 were cloned into a TA cloning vector. Sanger sequence analysis revealed that lines 5 and 11 are biallelic and contain four types of mutations: a 6-, 5- or 4-nt deletion, or an A insertion. Interestingly, in line 5, both alleles lost the *Bsr*I restriction site whereas in line 11, one of the alleles restored the *Bsr*I cut site after Cas9-mediated editing of the target (Fig. 2c).

T0 transgenic tomato lines were grown to maturity and self-pollinated to generate T1 progeny. T1 progeny from lines 1, 2, 5 and 11 were designated with a small letter next to the line number (1a, 1b, etc.). Genomic DNA was extracted from the T1 progeny and the target region of ^*CCD8*^Cas9 was amplified using PCR primers flanking the target. When this analysis was performed on T1 lines, results were similar to those with the T0 lines: lines 2a and 2b were completely digested, indicating that the *Bsr*I site was preserved; however, lines 1a, 1b, 5a, 5b, 5c and 11a gave PCR fragments that were resistant to *Bsr*I digestion, indicating that the CRISPR-generated mutations in these lines were inherited from the T0 lines (Fig. 2d). Sanger sequencing of the non-*Bsr*I-cut fragments showed deletion mutations of various lengths upstream of the PAM sequence, similar to T0. Lines 1a and 1b each contained the same 1-nt deletion and lines 2a and 2b each had the same 3-nt deletion. Moreover, biallelic mutations were detected in lines 5a, 5b, a 4-nt deletion was observed in line 5c, and a 6-nt deletion was found in line 11a (Fig. 2e). At least 15 plants from each of the T1 lines were examined for genotype at the target site using Sanger sequencing of target-site PCR products. All T1 plants from two T0 homozygous lines (T0-line 1 and T0-line 2 of ^*CCD8*^Cas9) were homozygous for the same mutations. In contrast, the biallelic T0 lines (T0-line 5 and T0-line 11 of ^*CCD8*^Cas9) of the Cas9-generated mutants were segregated in the T1 generation according to classic Mendelian genetics, and the ratios between the two mutations in a biallele were close to 1:2:1 as reported previously (Supplementary Table S1)^25^. The existence of the same mutations in sibling progeny suggested that the CRISPR mutation event occurred prior to meiosis in T0. Inheritance of the mutations in homozygous and biallelic T0 plants by T1 plants suggested that most, if not all, of the mutations resulting from genome editing activity are highly stable in nature and can be inherited in subsequent generations. Moreover, examination of transgenic region in some of the T1 generation plants suggest that 35% (5/14) of line 2 and 25% (4/16) of line 5, T1 plants were detected to be transgene free (Supplementary Fig. S2 and Table S1). The results indicated that ^*CCD8*^Cas9 targeted mutations inherited to next generation in transgene free plants. The putative off-target sites associated with ^*CCD8*^sgRNA were evaluated by CRISPR-P program using the ^*CCD8*^sgRNA sequence against the tomato genome^26^. We analyzed three potential off-targets sites with high scores, which occurred in the intergenic and CDS regions of the tomato genome. Two plants from each line were selected from the T1 generations of ^*CCD8*^Cas9-edited tomato plants. Sequencing of PCR products from these regions revealed no changes in the putative off-target sites in the ^*CCD8*^Cas9*-*mutant lines (Supplementary Fig. S3 and Table S2).

### Morphology and resistance to *P*. *aegyptiaca* infestation of ^*CCD8*^Cas9 mutated tomato plants

**Figure 3:**
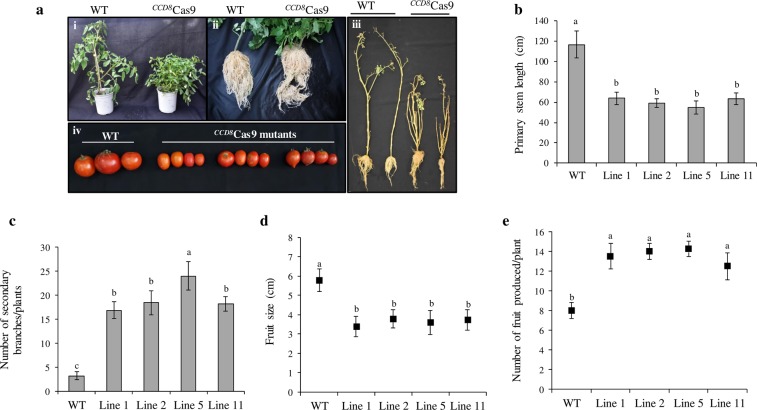
Morphological characterization of ^*CCD8*^Cas9 edited tomato lines. **(a–i)** WT- wild type plants with normal vegetative growth; ^*CCD8*^Cas9 T1 transgenic lines of the ^*CCD8*^Cas9-edited plants. **(a–ii)** Root morphology and **(a–iii)** plant height and branching of the wild type and ^*CCD8*^Cas9 mutant. **(a–iv)** Comparison of tomato fruits obtained from the different ^*CCD8*^Cas9 mutants in the T2 generation and wild-type plants. **(b)** Plant height after 3 month of growth, average ± SE (n = 11) (**c**) Quantitative estimate of secondary branches in 1 month old ^*CCD8*^Cas9 mutated tomato plants, average ± SE (n = 7) **(d)** mature fruit size, average ± SE (n = 15) and **(e)** number of fruit produced average ± SE (n = 6), in ^*CCD8*^Cas9 mutated lines and wild type plant. The different small letters not connected by same letter on each bar indicate statistically significant differences compared to wild-type plants (p-value < 0.05; Student’s t-test).

Previous studies on SL biosynthesis using rice dwarf mutants have reported that SLs regulate plant growth and morphological architecture^27^. Furthermore, *ccd8* a SL-deficient mutant of pea is known to exhibit increase in shoot branching, lateral roots and overall dwarfing^28^. We also observed similar phenotypic profile in ^*CCD8*^Cas9 mutated lines such as highly branched shoots, increased lateral roots, decreased shoot heights and reduced fruit sizes as compared to the wild type plants (Fig. 3a–c). Although, morphologically, all ^*CCD8*^Cas9 mutant lines showed highly branched shoots irrespective to the type of mutation but no significant differences were found in the root mass between ^*CCD8*^Cas9 mutated and control plants (Supplementary Fig. S4). Interestingly, ^*CCD8*^Cas9 mutated tomato lines produced considerable more number of fruits with reduced sizes as compared to the non-mutated wild-type plants (Fig. 3d,e).

**Figure 4:**
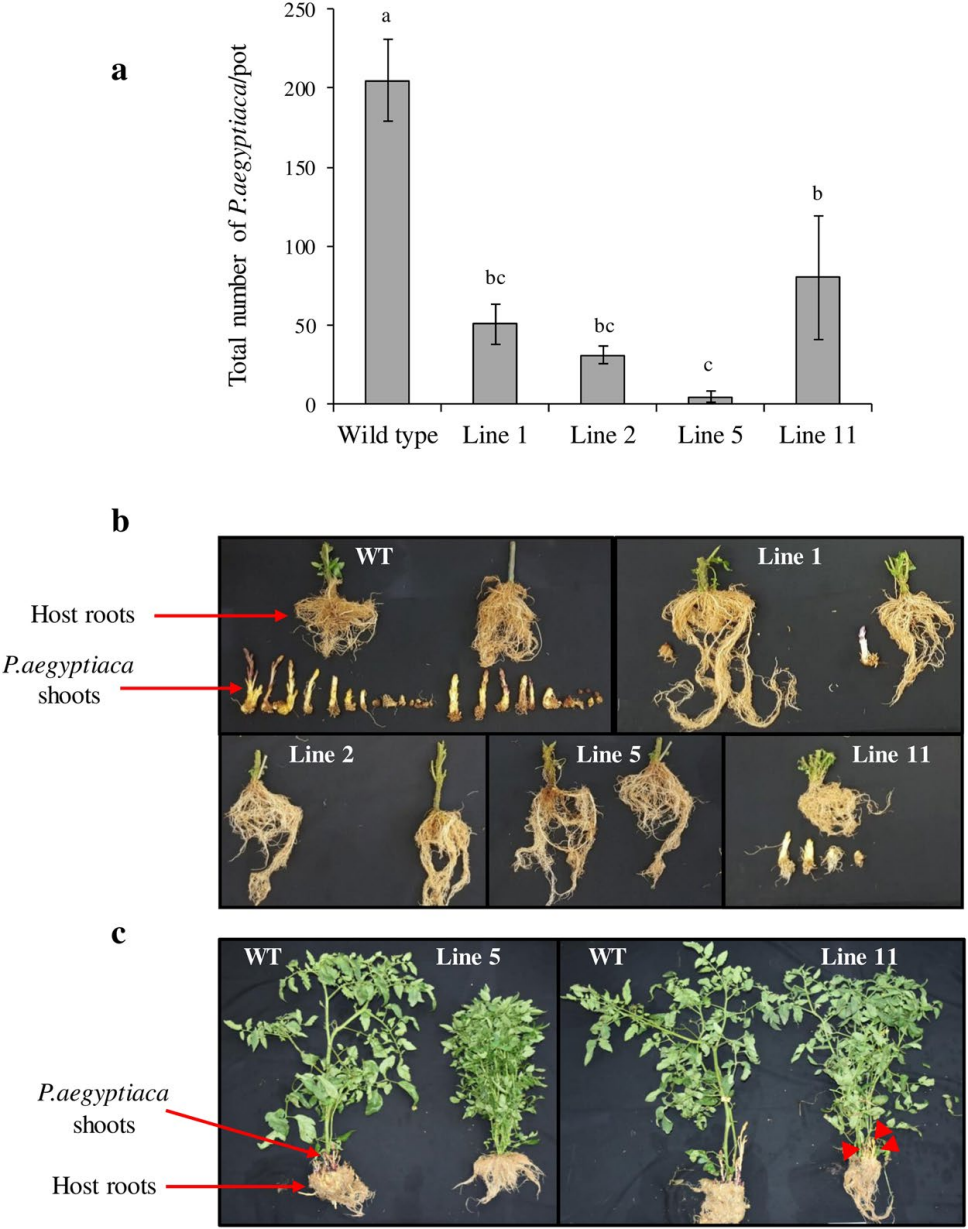
Resistance of tomato ^*CCD8*^Cas9-edited T1 lines to the parasite infestation as compared to the wild type. To evaluate host resistance to the parasite, host roots of the tomato wild-type (WT) and ^*CCD8*^Cas9 mutated T1 lines were rinsed after 3 months of infestation with *P*. *aegyptiaca* seeds. Tubercles larger than 2 mm in diameter were considered for analysis. **(a)** Number of *P*. *aegyptiaca* tubercles and shoots attached to the mutant and non-mutant tomato plants in the pot assay. Bars represent average of two experiments with four independent plants of each T1 line (irrespective of type of mutation, either homozygous or biallelic within the lines) ±SE values. **(b)** Representative images of parasite infestation of the host plant; WT shows number of parasites attached to the root while ^*CCD8*^Cas9 mutants have reduced parasite infection. **(c)** Whole plant images of mutant lines 5 and 11 showing the parasite shoots and tubercles developed on those roots as compared to the WT plants. Red triangle indicates *P*. *aegyptiaca* shoots developed on host plant line 11, while host plant line 5 is free of *P*. *aegyptiaca* infestation.

To analyze whether the CRISPR/Cas9-generated mutations in the *CCD8* gene confer resistance to *P*. *aegyptiaca*, independent transgenic tomato plants from T1 lines representing the ^*CCD8*^Cas9 knockout phenotypes, were triggered with *P*. *aegyptiaca* seeds. Randomly chosen T1 progeny of each lines, irrespective of their zygosity (homozygous or biallele) were transplanted into small pots containing soil infested with *P*. *aegyptiaca* seeds (20 mg/kg soil) and grown for 3 months in a greenhouse. Two separate experiments with four replicates per treatment were conducted. To measure the resistance of the ^*CCD8*^Cas9 mutated lines, we counted only fresh and viable parasite tubercles which are larger than 2 mm in diameter from each plant. The numbers of attached parasitic tubercles and shoots were significantly reduced in the ^*CCD8*^Cas9 mutated lines (1, 2, 5 and 11) relative to the wild-type plants. However, the decrease in *P*. *aegyptiaca* in some of the line 11 mutants was less pronounced relative to the wild-type plants than that observed for lines 1, 2 and 5 (Fig. 4a–c).

### Analysis of orobanchol content in the roots of ^*CCD8*^Cas9 mutated tomato lines

**Figure 5:**
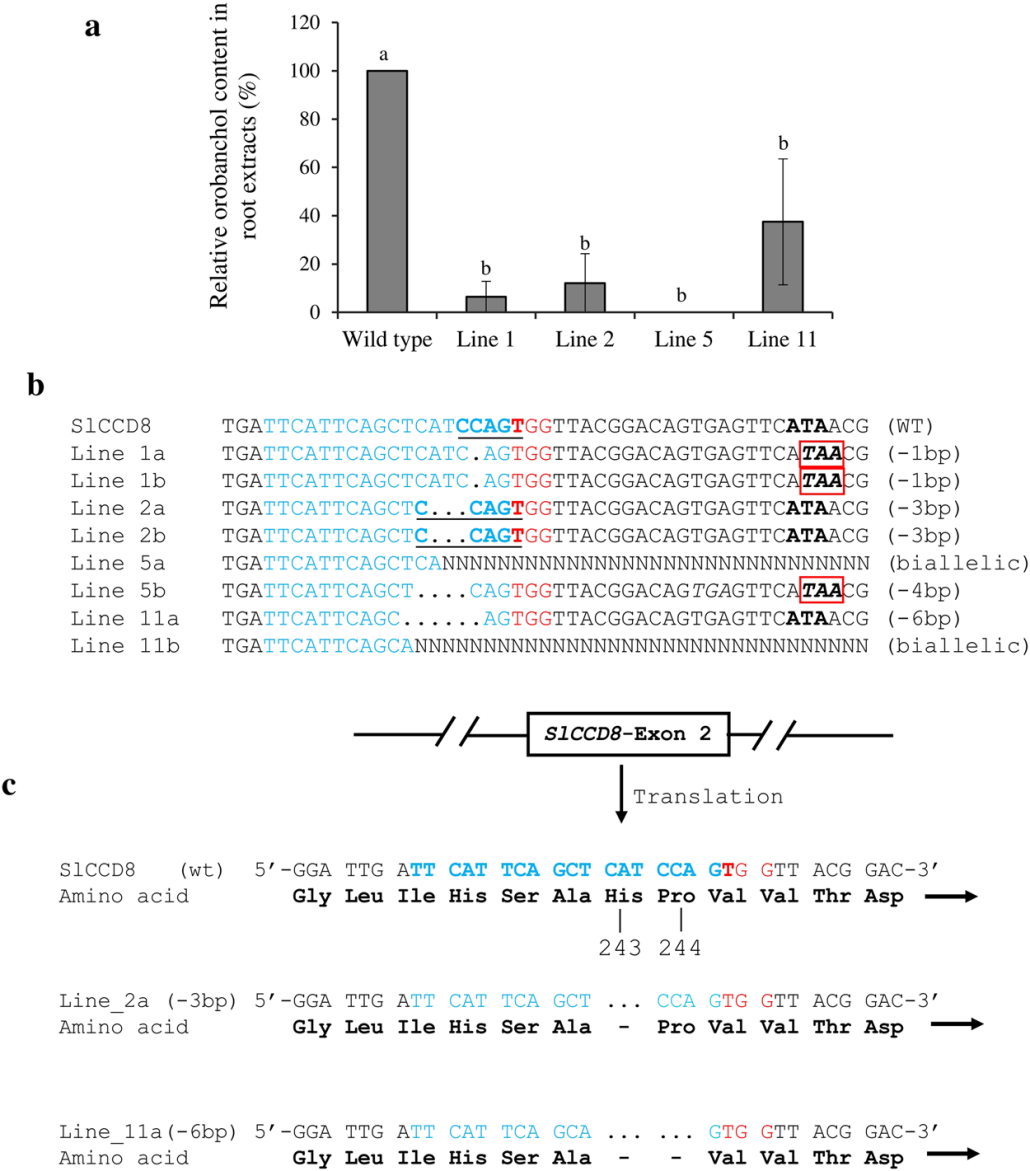
Orobanchol content, genotyping and amino acid sequence analysis of the ^*CCD8*^Cas9 mutated T1 plants. (**a)** Orobanchol contents in the roots of tomato ^*CCD8*^Cas9 edited lines (1, 2, 5 and 11) as compared to wild type. LC-MS/MS analysis was done with two independent biological samples from each line. Data presented as average ± SE. **(b)** PCR product sequence alignment of ^*CCD8*^Cas9 edited lines (1, 2, 5 and 11) used for LC–MS/MS analysis. PAM is shown in red; *Bsr*I site shown in bold underlined; DNA deletions are shown in black dots and deletion sizes (nt) are marked on the right side of the sequences; Nucleotide sequence inside red box encode for stop codon. **(c)** Amino acid sequences for line 2a (His-243 deletion) and line 11a (His-243 and Pro-244 deletion) compared to wild-type (WT) *CCD8* proteins.

The tomato host plant produces different kinds of SLs—mainly orobanchol, didehydroorobanchol isomer 1 and 2, and the aromatic SL solanacols, including the recently identified orobanchyl acetate, 7-hydroxyorobanchol isomers 1 and 2, and 7-oxoorobanchol^29^. Several studies have shown that SLs (a family of chemical molecules) play a critical role in the germination of parasitic weeds. However, *Orobanche* preferentially utilizes orobanchol as the most active germination stimulant (>80% germination), whereas  solanacol and 7-oxoorobanchol are weak stimulants^30^. To explore the connection between SL biosynthesis in the ^*CCD8*^Cas9 mutants and their resistance to *P*. *aegyptiaca* infection, we analyzed the total orobanchol content in the roots of wild-type and ^*CCD8*^Cas9 mutated T1 lines by LC–MS/MS. Orobanchol levels were significantly decreased in the ^*CCD8*^Cas9 mutated lines 1b, 2a, and 11b compared to the wild type, whereas orobanchol was not detectable in lines 1a, 2b, 5a, or 5c (Fig. 5a and Supplementary Table S3). This is consistent with line 5 showing the highest resistance to *P*. *aegyptiaca*. In addition, although *CCD8* was modified in line 11a, and the plant exhibited the typical dwarfing and shoot-branching phenotypes of reduced SL, its orobanchol content was higher than in the other modified lines. The higher orobanchol content was consistent with its lower resistance to *P*. *aegyptiaca*. To assess the higher orobanchol content in line 11a, we analyzed the DNA mutations and resulting amino acid sequences in all ^*CCD8*^Cas9 mutated lines that were sampled for LC–MS/MS analysis after PCR products ligated to the TA cloning vector (Fig. 5b and Supplementary Fig. S5). The type of DNA mutation and the amino acid sequence in line 11a demonstrated that only 2 amino acids, His-243 and Pro-244, were deleted due to Cas9 editing in the target *CCD8* gene, while the rest of the coding sequence was similar to the wild-type protein (Fig. 5c).

### ^*CCD8*^Cas9 mutation affects carotenoid content and its biosynthetic pathway

**Figure 6:**
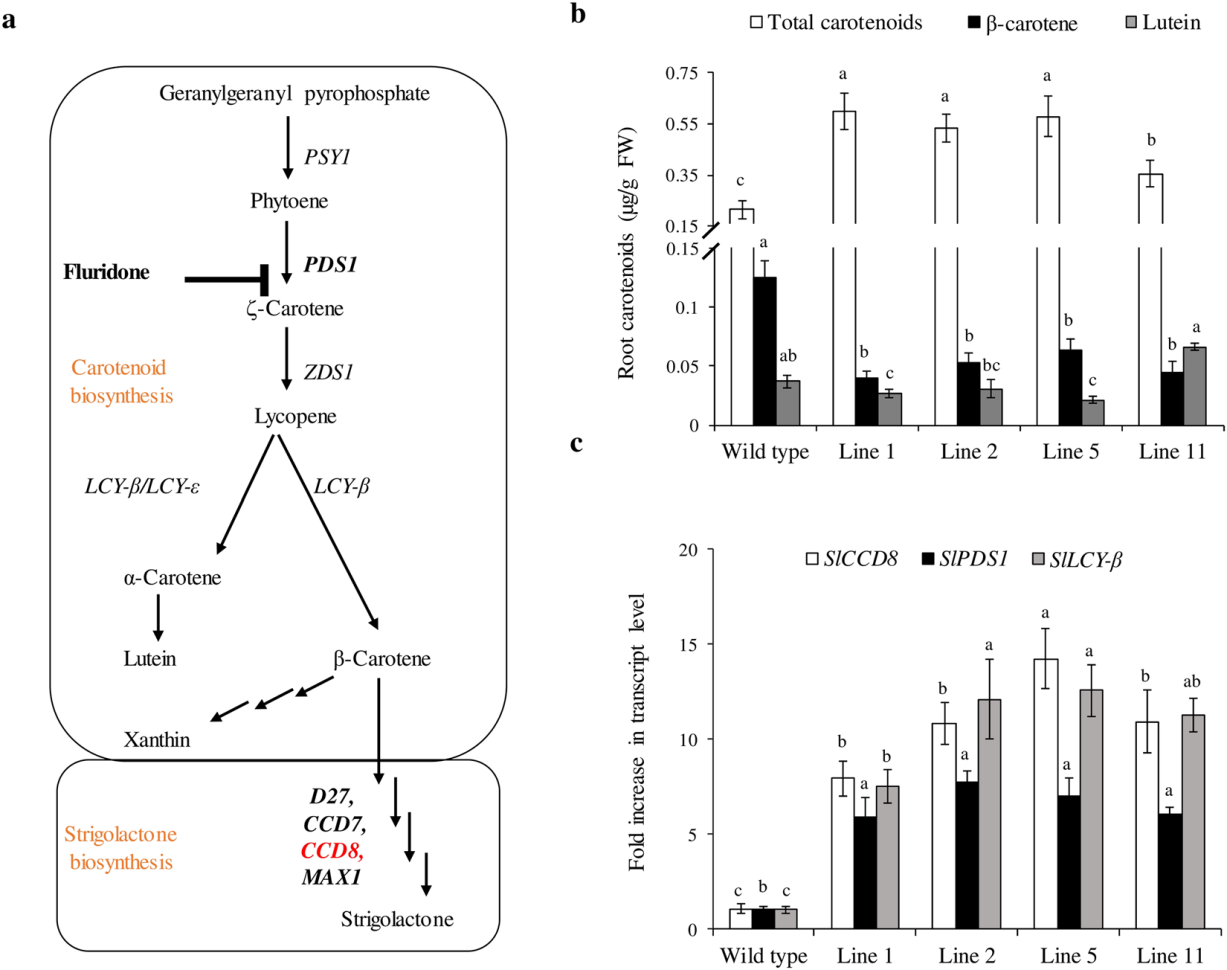
Carotenoid-biosynthesis pathway, carotenoid contents and transcript expression of three candidate genes (*SlCCD8*, *SlPDS1* and *SlLCY-β*) in ^*CCD8*^Cas9 mutants. (**a)** Schematic representation of carotenoid-biosynthesis pathway leading to β-carotene formation and strigolactone biosynthesis. Target site for fluridone shown by blunt black arrow. Enzymes are aligned with arrows. The enzymes belonging to carotenoid biosynthesis are: phytoene synthase (PSY1); phytoene desaturase (PDS1); ζ-carotene desaturase (ZDS1), lycopene ɛ-cyclase (LCY-ε); lycopene β-cyclase (LCY-β). The enzyme belongs to SL biosynthesis are *D27*, carotenoid cleavage dioxygenases 7, 8 and *MAX1*. **(b)** Carotenoid content in tomato roots of control and ^*CCD8*^Cas9 mutant plants. Values are based on the analysis of 3 months old plants grown in green house, bars represent the average of two experiments, was done with the pooled sample from two independent plant roots for each line ± SE. Statistical differences between the wild-type control were calculated with Student’s t-test (P < 0.05). Different small letter on bar indicate a significant difference between the ^*CCD8*^Cas9 edited lines and the WT plants. (**c**) Quantification of *SlCCD8*, *SlPDS1* and *SlLCY-β* transcript levels in roots of ^*CCD8*^Cas9 edited lines and wild type (WT) plants using quantitative real-time PCR. Expression level of transcript was displayed after normalization with internal control tomato elongation factor-1α (EF1-α). Bars with different letters are significantly different from each other (student t-test at p < 0.05 when compared with the controls). Results represent the average of at least three independent experiments with three technical repeat ± SE (n = 3), and expressed as fold increase in expression of transcripts relative to the control plants.

Carotenoid biosynthetic pathway derivatives all trans β- carotenoid leads to production of SL^31^. Since *CCD8* catalyze a key step in SL biosynthesis from carotenoids; hence, we are interested to discover whether ^*CCD8*^Cas9 mutation affects carotenoid content and its upstream biosynthetic pathway. A simplified scheme of the correlation between carotenoid and SL biosynthesis pathways and fluridone target site is illustrated in Fig. 6a. First, to explore whether ^*CCD8*^Cas9  mutation affect the carotenoid content, the content and type of carotenoid present in the root of wild type and ^*CCD8*^Cas9 mutated lines were analyzed by HPLC method. Interestingly, ^*CCD8*^Cas9 mutation altered the profile of different types of carotenoids and its derivative, such as total carotenoids, lutein; β-carotene were substantially altered from the wild type (Fig. 6b). To further gain insight into the above results, we analyzed the expression of prominent gene Phytoene desaturase-1 (*Solyc03g123760*) and Lycopene cyclase 1-β (*Solyc04g040190*), involved in the carotenoid biosynthetic pathway which acts upstream of *CCD8*. Results obtained using quantitative real-time PCR demonstrated that expression of *PDS1*, *LCY-β* and *CCD8* was upregulated in ^*CCD8*^Cas9 edited T1 lines as compared to the wild type (Fig. 6c).These results demonstrate that a decrease in SL content in the root of ^*CCD8*^Cas9 mutants, affect the carotenoid profile by modulating expression of the gene involved in carotenoid pathway.

## Discussion

Biotic stresses induced great economic challenges for farming and food production worldwide. Broomrapes (*Phelipanche* and *Orobanche* spp.) that affect the roots of many economically important agriculture crops throughout the semiarid regions of the world especially the Mediterranean and Middle East, are regarded as some of the most serious pests in vegetable and field crops. Effective means to control parasitic weeds are scarce and lack of novel sources of resistance limits our ability to manage newly developed, more virulent broomrape races. Therefore, an innovative solution to the problem is greatly needed.

Recent work utilized the power of CRISPR/Cas9 to engineer the rice plant architecture through genomic editing of *OsCCD7* gene, having decreased SL and reduced *Striga hermonthica* germination^32^. Utilizing similar CRISPR/Cas9 genome-editing strategy, we have developed non-transgenic tomato mutant plants with no “foreign-DNA”^33^ that exhibits resistance to *P*. *aegyptiaca*. We designed a ^*CCD8*^sgRNA construct to target the second exon of the tomato *CCD8* gene to disrupt SL biosynthesis. Several independent T0 transgenic tomato lines—1, 2, 5 and 11—were generated, of which lines 1 and 2 were homozygous mutants whereas lines 5 and 11 were biallelic in nature. In general, T0 mutants presented somatic mutations; to avoid this, T0 plants were self-pollinated to generate T1 homozygous plants. In the T1 generation, we found homozygous deletions and insertions in the target gene that were biallelic in the T0 plants (lines 5 and 11) without any new mutation detected. T1 lines 1, 2, 5 and 11 were selected for further analysis because of their stable genetic modification. The different types of mutations observed in the different lines may be due to the differential activity of Cas9, depending on the transgene insertion site, as shown previously in tomato^34^. In addition, Sanger sequencing of potential off-target sites with mismatches of less than 4nt with ^*CCD8*^sgRNA did not identify any mutations. The CRISPR/Cas9 system has emerged as a powerful gene-editing tool and has been successful in more than 20 crop species to date. The heritability of the mutated genes and the generation of transgene-free plants are of major concern when using the CRISPR/Cas9 system^35^. To follow heritability, the gene *PDS1* was used to demonstrate the inheritance of mutations induced in *Solanum lycopersicum*^36^. Those authors showed that the CRISPR/Cas9 can efficiently induce heritable mutations in tomato plants from the T0 to T2 generation, and that homozygous and biallelic mutants are generated, in the first generation. In our study, we also showed that the ^*CCD8*^Cas9 mutations induced in T0 lines are inherited by the T1 generation.

Previous reports on the morphology of tomato *SlCCD8* knock down plants by gene silencing have shown an increase in shoot branching, altered lateral adventitious root growth and decrease in plant height^37^. Similarly another studies on SL biosynthetic gene *SlCCD7*, demonstrates the *SlCCD7* antisense tomato lines also display increased branching, reduced SL content and significantly decreased germination rate of *O*. *ramosa*, however no changes in carotenoid content in the roots were observed^38^. Our results were partially consistent with the previous studies, we observed similar phenomenon in the ^*CCD8*^Cas9 tomato plants. Mutated-plants displayed a dwarf phenotype, an increased number of shoot branches, and an increased number of adventitious roots compared to the wild-type plants. In contrast to the previous report, here we discovered that ^*CCD8*^Cas9 mutants have altered carotenoid content and differential expression of genes involved in carotenoid biosynthetic pathway. An explanation for the contradictory results could be due to the differences between mechanisms of siRNA and CRISPR/Cas9 systems. We hypothesize that absence of *CCD8* gene followed by mutation in tomato plants using CRISPR system, will not restore feedback regulation. However, a small percentage of functional *CCD8* gene that could escape gene-silencing system will provide feedback regulation and restore the carotenoid level upstream in the carotenoid pathway.

To determine host resistance to plant parasite *P*. *aegyptiaca*, we used a pot system containing soil infested with *P*. *aegyptiaca* seeds. In the current study, concomitant with the decrease of SL content, there were significant decreases in the number of parasite tubercles and shoots growing on tomato lines 1, 2, 5 and 11 (Fig. 4) relative to the control treatment was observed. Line 5 exhibited the highest resistance to *P*. *aegyptiaca* infestation. In contrast, the non-mutated wild-type plants were highly susceptible to the parasite infestation.

To date, several unique SLs, including orobanchol and solanacol, have been reported in the root exudates and extracts of tomato (*Solanum lycopersicum*). Since orobanchol is a major SL in tomato root exudates^39^, and is a specific germination stimulant for *P*. *aegyptiaca*, we determined orobanchol content in the roots of ^*CCD8*^Cas9 mutant lines. Orobanchol levels were found to be significantly decreased in the ^*CCD8*^Cas9 mutated lines (1, 2 and 11) relative to the wild type. Interestingly, orobanchol was not detectable in line 5, which was shown to be the most resistant to *P*. *aegyptiaca* infestation. The higher orobanchol content and only partial resistance to *P*. *aegyptiaca* obtained in line 11a (Figs 4a and 5a) could be explained by either the deletion of the cyclic amino acid Pro-244 which is involved in the protein’s structural and conformational rigidity, can partially restore protein functionality or imbalanced carotenoid profile still provide support in line 11 to produce SL. However, in line 2a, the deletion of 1 amino acid (His-243) strongly affected *CCD8* activity, as reflected by significant decreases in orobanchol content and *P*. *aegyptiaca* infestation.

Our study also reports that ^*CCD8*^Cas9 mutated lines have increased transcript levels of *PDS1*, *LCY-β* and altered carotenoid contents. This could be due to the decrease in SL content positively affecting the feedback regulation of these upstream biosynthetic-pathway genes. Another study showed that when the carotenoid-biosynthesis pathway gene *PDS1* is inhibited by fluridone treatment, tomato seedlings show a strong decrease in SL content and less germination of *P*. *ramosa* compared to the wild-type treatment^39^. These results indicate that inhibition of the carotenoid-biosynthesis pathway not only affects carotenoid biosynthesis but is also involved in modulation of downstream SL pathways and vice versa. Hence, there is a tight correlation between SL production, carotenoid biosynthesis and the resistance mechanism.

Previous studies demonstrated that SL-biosynthesis pathways are strictly controlled through negative-feedback inhibition such as *D10* (*CCD8* orthologs in rice) transcript levels are elevated in the enhanced-branching *d10-1* dwarf mutants of rice and *RMS1* (*CCD8* orthologs in pea) transcript level increased dramatically in *ramosus* mutants of pea and treatment with SL analog GR24 leads to restore the transcripts level^40,41^. Based on these results, we expected changes in the expression of *CCD8* transcripts in our mutants. To measure *CCD8* expression, we analyzed its relative transcript abundance using quantitative real-time PCR. Interestingly, the *CCD8* transcript level was substantially increased in the transgenic ^*CCD8*^Cas9 mutated tomato plants (lines 1a, 2a, 5a and 11a) relative to the wild-type plants (Fig. 6c). In addition to decrease in orobanchol content, line 5 had shown highly increase in the transcript level in comparison to line 1, 2 and 11, which are negatively regulated by SL, provide a basis to explain its higher resistance against *P*. *aegyptiaca* compared to other lines.

Similar results have been also reported by others, where expression of the SL-biosynthesis gene in *Arabidopsis* was upregulated in SL-biosynthesis-defective mutants (*max1*, *max3* and *max2*), suggesting that SL content influences the transcript level of genes involved in SL biosynthesis^42^. Another study found increased transcript level of *SlD27* and *SlCCD8* in tomato roots after *P*. *ramosa* infection, suggesting activation of the SL-biosynthesis pathway upon interaction of plant parasite with host root^43^.

In our previous studies, we showed that production of siRNA molecules in tomato targeting *P*. *aegyptiaca* genes facilitates suppression of the parasite genes and results in enhanced resistance to the parasite^44^. Intriguingly, in a recent study, SL-deficient tomato lines (*SlCCD8*-RNAi lines) displayed increased tubercle development and enhanced infestation with pre-germinated *P*. *ramosa* seeds^45^. We assume that the contrast with our results is due to difference in mechanism of germination and susceptibility. In contrast to previous study, we do not pre germinate the parasite seeds, we directly triggered SL-deficient tomato lines with the parasite *P*. *aegyptiaca* seeds because *P*. *aegyptiaca* seeds become SL independent upon pre-germination. Another possible explanation for the increased parasitic infection in *SlCCD8*-RNAi lines, might be their enhanced auxin transport or altered auxin levels, as has been reported for the *Arabidopsis* SL-deficient mutant *max4*^46^.

In this study, CRISPR/Cas9 was used to precisely knock out the *CCD8* gene in tomato. Disease resistance based on the editing of specific genes can be easily applied to other susceptible crops without long-term backcrossing, and even those that are distantly related. This method is expected to be effective against other *Phelipanche* and *Orobanche* species, if the parasite species share sufficient homology in their target sequences. In addition, the mutated plants are devoid of foreign DNA sequences and are not considered genetically modified organisms.

One of the significant limitations of the CRISPR/Cas9 system is the generation of significant off-target cleavage as a result of non-specificity of the sgRNA with mismatched complementary target DNA within the genome. Thus, that may alter the function of a gene and may result in genomic instability. Although, several modifications of the Cas9 enzyme and sgRNA designing methods have been developed to increase target specificity, the off-target cleavage is still considered as major limiting factor with CRISPR/Cas9 technology^47^. Additionally, under regulatory regimes in many countries, the new breeding technology CRISPR/Cas9 does not fall under the definition of a GMO, but in the EU countries CRISPR/Cas9 is still considered as GMO^48^.

In conclusion, using CRISPR/Cas9, we generated an effective mutation in tomato to be executed in breeding programs for resistance against parasitic weeds. In the current study, we demonstrate that genetic resistance to parasitic weeds can be created using CRISPR/Cas9. We used this technology to edit *CCD8* (a SL-biosynthesis gene) in tomato. We chose tomato due to its high economic importance and high susceptibility to parasitic weeds. We chose to knock out the *CCD8* gene due to its role in SL production and its clear morphological phenotype (increased shoot branching, adventitious root formation and dwarfing). We realize that mutations in *CCD8* can negatively affect tomato plant morphology; however, to avoid this issue, our system could be used in a rootstock grafted to a wild-type scion, and might be combined with tomato rootstocks that are already resistant to fungal pathogens, viruses, and nematodes. Further study is needed with grafting experiments to investigate this concept, which might be applicable to a wide range of crop plants. To the best of our knowledge, this study is the first to report the generation of tomato plants that are resistant to the weedy parasite *P*. *aegyptiaca* using the CRISPR/Cas9 genome-editing technology.

## Experimental Procedures

### Materials and growth conditions

Tomato seeds (*Solanum lycopersicum L*.) T5 strain and its derivatives were used throughout the experiments. Tomato seeds were surface-sterilized and grown on half strength Murashige and Skoog (MS) basal medium (CAISSON Laboratories®, USA) containing 1.5% sucrose (Sigma), pH-5.8 and 7 g/L phytagel (Sigma). The parasite seeds were collected from infected field in Northern Israel and used to infest tomato host plants. For PCR product cloning, we used pGEM^®^-T kit from Promega.

### ^CCD8^sgRNA design and plasmid construction

The tomato Carotenoid Cleavage Dioxygenase 8 (*CCD8*), (*Solyc08g066650*) was chosen as the target gene for editing. For the delivery of the Cas9/^*CCD8*^sgRNA we used pMR290 that was modified from pDe_Cas9^49^. In this vector the *EcoR*I site that is in the gateway cassette was eliminated by site directed mutagenesis following by removing the Phosphinothricin (PPT) selection cassette by digestion with *Hind*III and fill-in. A new *Hind*III site was introduced upstream of the PcUbi promoter in which, a new selection cassette (Nos-p-NptII-Nos-ter) was introduced. The PcUbi promoter driving the Cas9 was replaced with 2 × 35 SΩ promoter (CaMV 2 × 35S promoter with omega enhancer) amplified from pICH47742^50^. The Pea3A terminator of the Cas9 cassette was replaced by AtHSP18.2 terminator. Target guides were designed using the CRISPRScan web tool^51^. Two complementary oligos with 4 bp overhangs (^*CCD8*^sgRNA-F and ^*CCD8*^sgRNA-R) were annealed and inserted into *Bbs*I digested pEn_Chimera^49^ using T4 ligation. The gRNA cassette was combined into pMR290 using LR II clonase reaction (Thermo Fisher) to generate the pENTR- ^*CCD8*^sgRNA:pMR290/Cas9 plasmid. The construct was transformed into tomato cultivar T5 by the University of California Davis Plant Transformation Facility using *Agrobacterium tumefaciens* strain EHA105.

### Mutant verification and genotyping

Tomato leaves and roots genomic DNA was extracted using a GeneJET plant genomic DNA purification mini kit and the genomic DNA flanks containing the target sites were amplified using the specific primers SlCCD8-Int-F and SlCCD8-Int-R. 300 ng of PCR products were digested with *Bsr*I enzyme (NEB, USA) at 65 °C for 16 hours in a PCR machine and then run on a 2% agarose gel using electrophoresis. For genotyping the plants, PCR products amplified with the above primers were directly sequenced after dilution using SlCCD8-Int-F primer. The primers used in this study are presented in Table S4.

### Analysis of off-target mutations

The potential off-target sites associated with ^*CCD8*^sgRNA target sequence were analyzed with the CRISPR-P program^26^. Three sites with the highest off-target probability score were selected. The genomic sequence flanks upstream and downstream to the off-target sites (300–400 bp) was amplified using off target site specific primers. PCR products were directly sequenced using Sanger sequencing.

### Evaluation of *P*. *aegyptiaca* resistance assay

Resistance of ^*CCD8*^Cas9 mutant tomato lines to the parasite were assayed as reported earlier^44^. For parasitic infection one-month-old tomato seedlings were transferred into pots containing soil with a peat moss to perlite mixture ratio of 3:1, infested with *P*. *aegyptiaca* seeds (20 mg/kg soil) and grown in a greenhouse under natural light with an average 14 h of daylight and a temperature of 20 ± 6◦C. Host roots from control and ^*CCD8*^Cas9 mutant plants were collected 90 days after exposure to the *P*. *aegyptiaca* seeds. The number of *P*. *aegyptiaca* tubercles larger than 2 mm diameter attached to host root were counted and photographed.

### SL extraction and analysis using LC-MS/MS

The SL extraction and quantification was performed as described previously^52^. Lyophilized tomato roots were ground into fine powders using liquid nitrogen and extracted with the ethyl acetate extraction method. The tissues were transferred to a 4–10 times volume of ethyl acetate. The flask was treated with an ultra-sonic bath for a few minutes and then placed in a cool place (4 °C) for 2–3 days. The tissues are filtered off and washed well with ethyl acetate. The combined ethyl acetate was washed with 0.2 M K_2_HPO_4_ or saturated NaHCO_3_ to remove acidic compounds. The extract was dried over anhydrous MgSO_4_ or Na_2_SO_4_, filtered and the solvent was evaporated under reduced pressure. Samples were dissolved in 2 ml acetonitrile/methanol and the quantification of orobanchol in extracts was performed using LC-MS/MS using suitable standard.

### RNA isolation and quantitative real-time PCR

Total RNA from tomato roots was extracted using spectrum plant total RNA kit (Sigma- STRN50-1KT) according to the manufacturer’s protocol. 500 ng of total RNA was used to obtained cDNA according to the protocol of Verso cDNA Synthesis Kit (Thermos Fisher Scientific). Quantitative real-time PCR (qRT-PCR) was performed in a volume of 10 μl using PerfeCTa® SYBR® Green FastMix®, ROX™ (Quanta biosciences) with 5 times diluted cDNA as template. Tomato elongation factor 1-α was used as an internal reference gene. Specificity of the primers was confirmed by melting curve analysis. The generated Ct values of target genes were normalized to the Ct value of housekeeping EF1-α gene. Relative expression was calculated using 2^−ΔΔCt^ method and expressed as fold increase with respect to control^53^.

### Carotenoid extract analysis

Carotenoid extract analysis was performed as reported with slight modifications^54^. In brief, 1.5 g of fine grinded roots was used for extraction in presence of 8 ml hexane: acetone: ethanol (50:25:25 v/v), followed by 5 min of saponification in 1 ml of 8% (w/v) KOH. After addition of 1 ml of NaCl (25%), the saponified material was extracted twice with hexane, which was then evaporated in speed vacuum. The solid pellet was resuspended in 400 μl of ACN: MeOH: DCM (45:5:50 v/v) and passed through a 0.2μm Nylon filter before HPLC analyses. Two independent biological samples from each line were pooled for carotenoid extraction analysis before HPLC.

### Statistical analysis

All the experiments were repeated independently for at least three times and statistical significance difference between mutants and control plants was analyzed by Student’s t-test using JMP Pro 14 software. A p-value of <0.05 was considered statistically significant that was marked with different letters.

## Supplementary information

Supplementary Information
