# Supplementary material for: CRISPR/Cas9-mediated mutagenesis of CAROTENOID CLEAVAGE DIOXYGENASE8 in tomato provides resistance against the parasitic weed Phelipanche aegyptiaca
Source: Sci Rep. 2019 Aug 7;9:11438. doi: 10.1038/s41598-019-47893-z (PMC6685993; doi:10.1038/s41598-019-47893-z)
Supplement: Supplementary file 1 — Supplementary Information [file 41598_2019_47893_MOESM1_ESM.pdf]

Supplementary Information:

CRISPR/Cas9-mediated mutagenesis of *CAROTENOID CLEAVAGE DIOXYGENASE 8* in tomato provides resistance against the parasitic weed *Phelipanche aegyptiaca*

Vinay Kumar Bari[1], Jackline Abu Nassar[1], Sally Marzouk Kheredin[1], Amit Gal-On[2], Mily Ron[3], Anne Britt[3], Daniel Steele[4], John Yoder[4] and Radi Aly[1*]

*Correspondence (email radi@volcani.agri.gov.il)

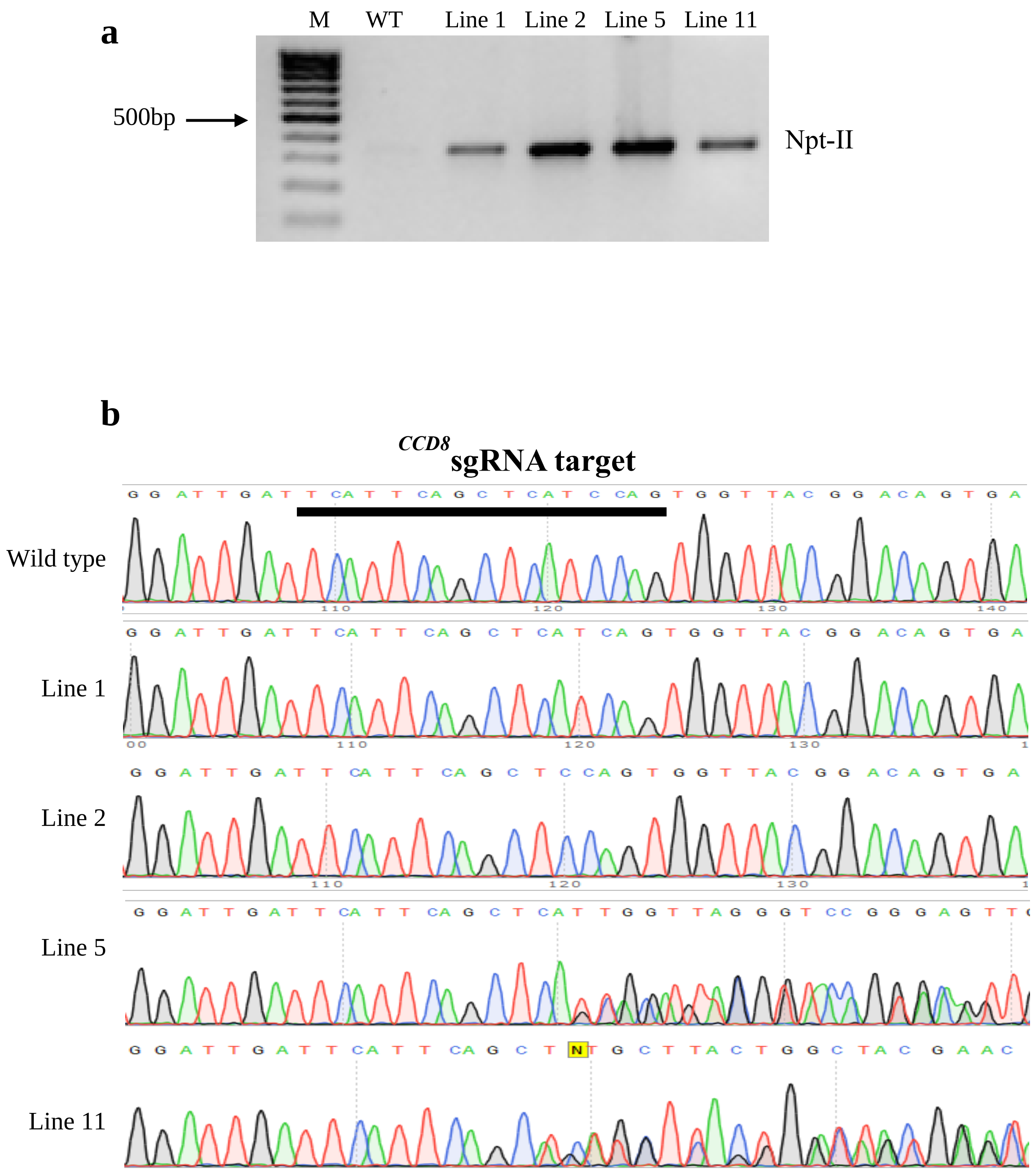

Supplementary Fig. S1. (a) Presence of transgene specific marker Npt-II in T0 generation of ^{CCD8}Cas9 edited lines. M: 100bp ladder **(b)** PCR product sequencing chromatogram of ^{CCD8}Cas9 edited T0 line tomato plants. Sequence underlined indicate ^{CCD8}sgRNA targeting sites. Wild-type sequence were used as the control.

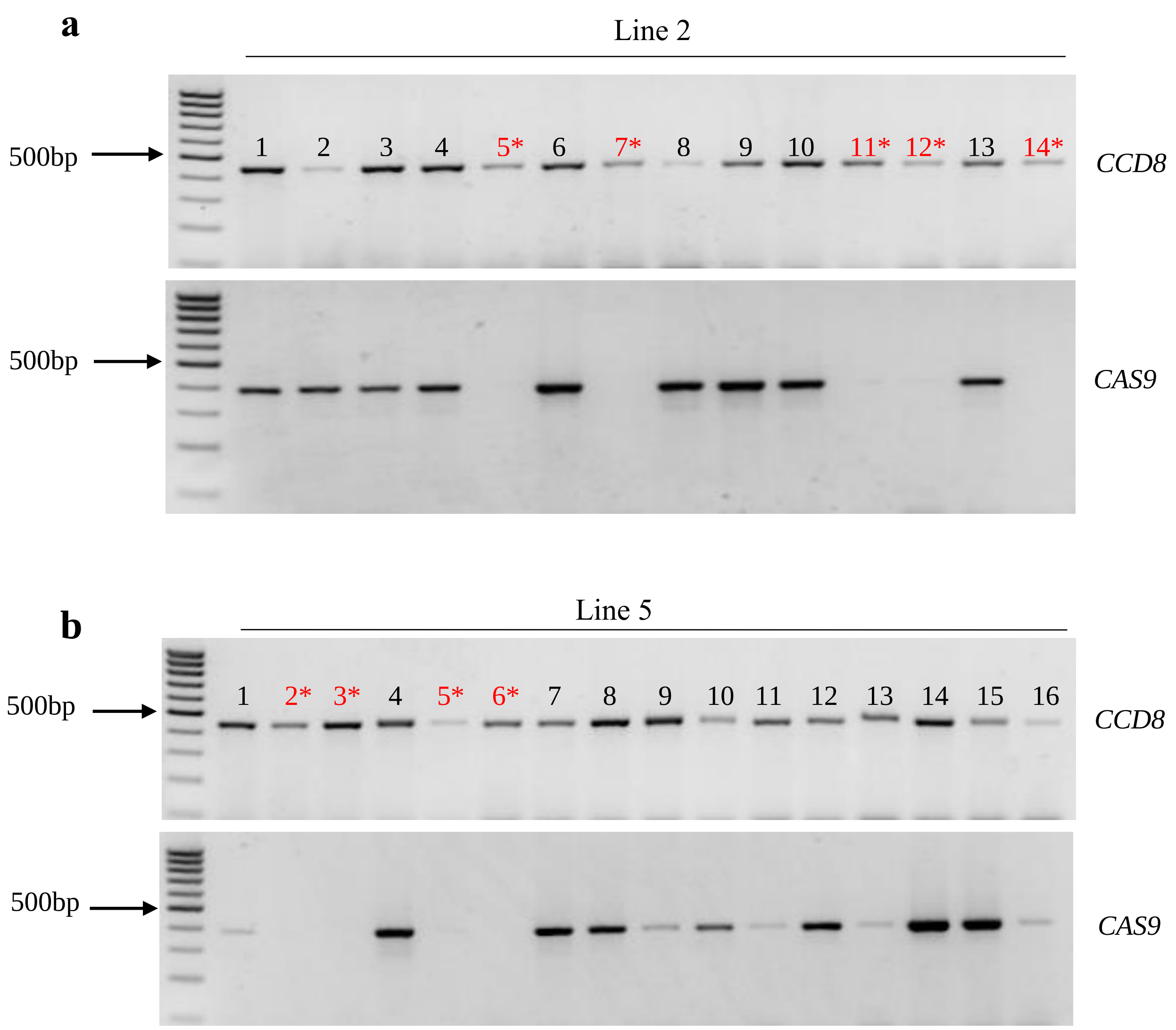

Supplementary Fig. S2. Identification of transgene free plants using Cas9 specific primer in T1 generation using different ^{CCD8}Cas9 mutants from Line 2 (a) and Line 5 (b). Red color number with asterisk denote transgene (Cas9) free plants.

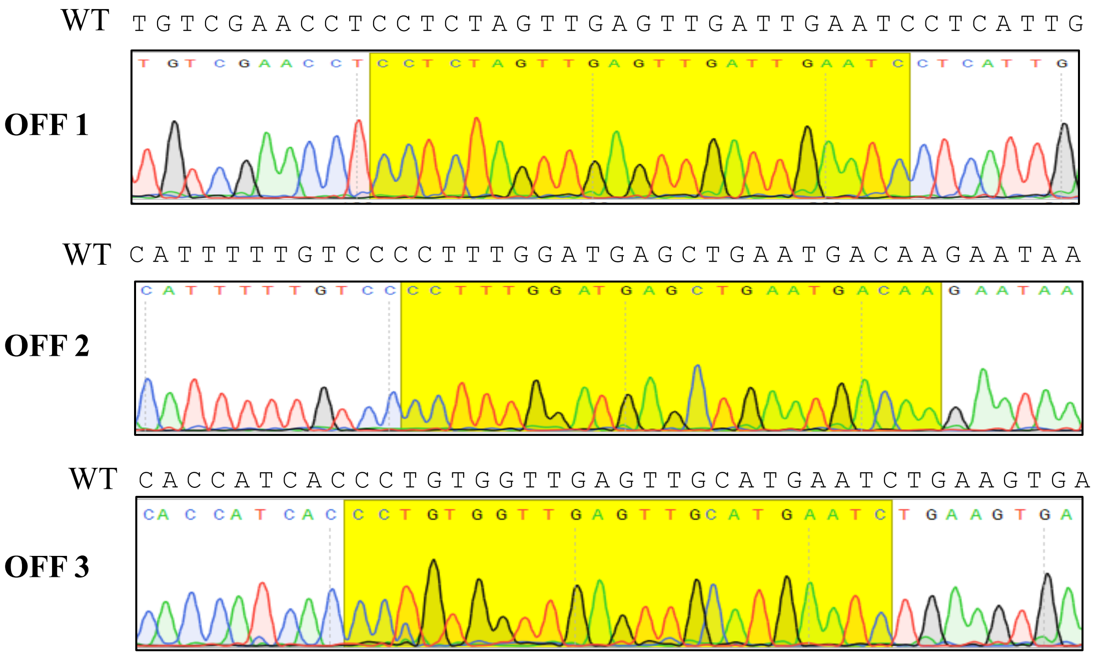

Supplementary Fig. S3. PCR product sequence chromatogram of potential off- target of the ^{CCD8}sgRNA. No off-target mutation detected in the genome of ^{CCD8}Cas9 edited lines. Region highlighted in yellow correspond to off target sites. For sequencing PCR product, off target specific reverse primer were used.

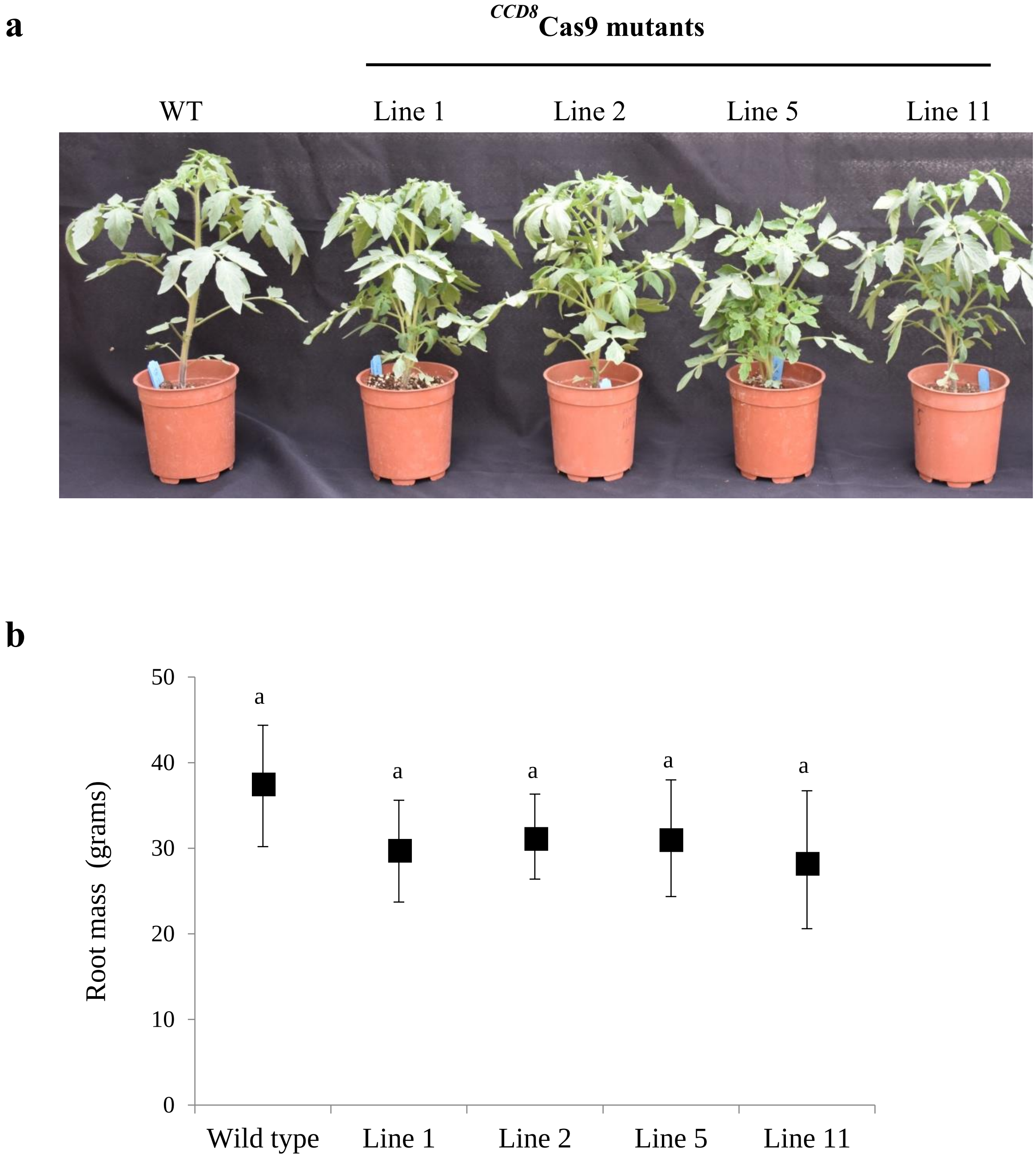

Supplementary Fig. S4. (a) Characteristic shoot branching phenotype of ^{CCD8}Cas9 knockout mutants in 1-month-old plants grown in green house. **(b)** Dry root mass of wild type and ^{CCD8}Cas9 mutant plants after 3 months of transplantation. Values are the average ± SD (n=8). (Level connected by same letters are not significant, $p < 0.05$; Student's t test).

```
SlCCD8          TGATTCATTCAGCTCATC.CAGTGGTTACGGACAGTGAGTTCATAACG (WT)
L5a-clone 1     TGATTCATTCAGCTCATCACAGTGGTTACGGACAGTGAGTTCATAACG (+1bp)
L5a-clone 2     TGATTCATTCAGCTCATCACAGTGGTTACGGACAGTGAGTTCATAACG (+1bp)
L5a-clone 3     TGATTCATTCAGCT.....CAGTGGTTACGGACAGTGAGTTCATAACG (-4bp)
L5a-clone 4     TGATTCATTCAGCT.....CAGTGGTTACGGACAGTGAGTTCATAACG (-4bp)
L5a-clone 5     TGATTCATTCAGCTCATCACAGTGGTTACGGACAGTGAGTTCATAACG (+1bp)
L5a-clone 6     TGATTCATTCAGCT.....CAGTGGTTACGGACAGTGAGTTCATAACG (-4bp)
L5a-clone 7     TGATTCATTCAGCTCATCACAGTGGTTACGGACAGTGAGTTCATAACG (+1bp)
L5a-clone 8     TGATTCATTCAGCT.....CAGTGGTTACGGACAGTGAGTTCATAACG (-4bp)

SlCCD8          TGATTCATTCAGCTCATCCAGTGGTTACGGACAGTGAGTTCATAACG (WT)
L11b-clone 1    TGATTCATTCAG......CAGTGGTTACGGACAGTGAGTTCATAACG (-6bp)
L11b-clone 2    TGATTCATTCAG......CAGTGGTTACGGACAGTGAGTTCATAACG (-6bp)
L11b-clone 3    TGATTCATTCAG.....CCAGTGGTTACGGACAGTGAGTTCATAACG (-5bp)
L11b-clone 4    TGATTCATTCAG.....CCAGTGGTTACGGACAGTGAGTTCATAACG (-5bp)
L11b-clone 5    TGATTCATTCAG......CAGTGGTTACGGACAGTGAGTTCATAACG (-6bp)
L11b-clone 6    TGATTCATTCAG.....CCAGTGGTTACGGACAGTGAGTTCATAACG (-5bp)
L11b-clone 7    TGATTCATTCAG.....CCAGTGGTTACGGACAGTGAGTTCATAACG (-5bp)
L11b-clone 8    TGATTCATTCAG......CAGTGGTTACGGACAGTGAGTTCATAACG (-6bp)
```

Supplementary Fig. S5. Sanger sequencing of ^{CCD8}Cas9 target region amplified from T1 generation of biallelic mutants of line 5 and 11 after cloning in pGEM-T vector, sequencing was done with M13-F Primer. Nucleotide sequence inside red box encode for stop codon.

Supplementary Table S1. Segregation pattern of ^{CCD8}Cas9 mediated targeted mutation from T0 to the T1 generation. The zygosity of homozygote and biallele in T0 plant lines were putative. +, Foreign DNA (Cas9) was detected; -, Foreign DNA (Cas9) was not detected.

^{CCD8}sgRNA/Cas9 Line	T0 generation		T1 generation		
	Mutation type	Zygosity	Mutation type/ total test	Mutation exist no./ total test (%)	Foreign DNA (Cas9) segregation (%)
Line 2	-3nt	Homozygous	-3nt(14/14)	14/14 (100%)	9+; 5- (35%)
Line 5	-4nt/+1nt	Bi-allelic	-4nt (6/16),+1nt (4/16),<br>-4nt/+1nt (6/16)	16/16 (100%)	12+; 4- (25%)

Supplementary Table S2. Mutations analysis of potential off-target sites in *CCD8*sgRNA edited T1-lines. To analyse the off target using CRISPR-P, high score value was considered as top hit. The PAM motif (NGG) is marked by red; mismatching bases are shown in blue colour.

**Name of putative off -target sites & score**	**Putative off-target locus**	**Sequence of the Putative off-target site**	**Number of mismatching bases**	**Presence of mutation detected**
OFF1 (0.511)	SL2.50ch05:-35934063 Intergenic	GATTCAATCAACTCAACTAG**AGG**	4	0
OFF2 (0.341)	SL2.50ch07:-56479727 Intergenic	TTGTCATTCAGCTCATCCAA**AGG**	4	0
OFF3 (0.286)	SL2.50ch12:-7245716 CDS (*Solyc12g017700.1*)	GATTCATGCAACTCAACCAC**AGG**	4	0

Supplementary Table S3. Orobanchol content in the roots of host plants using different ^{CCD8}Cas9 edited tomato T1-lines. Analysis of orobanchol in the root extracts was conducted by comparing retention time and mass transition with available orobanchol standards. Experiment was repeated two times, Data presented are from a typical single experiment. #ND (not determined)

^{CCD8}sgRNA/Cas9 edited tomato lines	Relative intensity of the chromatographic peaks area (Orobanchol content ppb)
Line 1a	ND#
Line 1b	1.8
Line 2a	3.4
Line 2b	ND#
Line 5a	ND#
Line 5c	ND#
Line 11a	8.9
Line 11b	1.6
Wild type	14.0

Supplementary Table S4. List of primers used in this study

**Oligo's name**	**Sequence (5' ⟶ 3')**	**Use**
^{CCD8}sgRNA-F	ATTGGTTCATTCAGCTCATCCAG	SlCCD8 Exon-2 targeting sgRNA cloning in Cas9 expression vector
^{CCD8}sgRNA-R	AAACCTGGATGAGCTGAATGAA	
SlCCD8-Int-F	CTGGAGTCGTTAAACTTGGGGATG	Mutation detection and sequencing
SlCCD8-Int-R	GCCACTGGCTTTACACATAACATGTAC	
SlCCD8-qF	CCAATTGCCTGTAATAGTTCC	qRT-PCR
SlCCD8-qR	GCCTTCAACGACGAGTTCTC	
SlPDS1-qF	TGATTATCCAAGACCAGAGCTG	
SlPDS1-qR	GCACCAGCAATAACAATCTCC	
SlLCYβ-qF	TTGACTTAGAACCTCGTTATTGG	
SlLCYβ-qR	AACAGTTCCCTTTGTCATTATCTC	
SlEFα1-qF	GATTGGTGGTATTGGAACTGTC	
SlEFα1-qR	AGCTTCGTGGTGCATCTC	
Npt-II-F	TCTTGTCGATCAGGATGATC	Transgene validation
Npt-II-R	AGAAGAACTCGTCAAGAAG	
hCas9-F	TAAGGGTGCTAGTGCTCAGTC	
hCas9-R	CGTTTTCCTCGTTATCCAAG	
OFF1-5F	AGAGAACAAGGCGAAGACAG	off target PCR and sequencing analysis
OFF1-5R	CTTGACCAAGTGCGTCTAAC	
OFF2-7F	GGATTTTATGTTTTGAGTTAGACC	
OFF2-7R	AAAGTAACAACAAAATCCAAAG	
OFF3-0F	CCTGTTTGGGTTGATAAAAG	
OFF3-0R	TCAGGAGTAGGCTCAAATATC	
M13-F	GTTTTCCCAGTCACGACG	Sequencing of pGEM-T cloned PCR product
